# Males and females have similar neuromuscular coordination strategies of the quadriceps during fatiguing repeated all-out cycling

**DOI:** 10.3389/fspor.2023.1248303

**Published:** 2023-09-15

**Authors:** Lauren A. Cederbaum, SangHoon Yoon, Julie N. Côté

**Affiliations:** Department of Kinesiology and Physical Education, Biomechanics of Occupation and Sport Laboratory, McGill University, Montreal, QC, Canada

**Keywords:** sex differences, repeated sprints, electromyography, muscle coordination, fatigue

## Abstract

**Introduction:**

An imbalance of vastus medialis (VM) and vastus lateralis (VL) muscle activation and patterns of dyscoordination may contribute to the sex discrepancy in the incidence of patellofemoral pain syndrome (PFPS). While some studies have examined sex-specific VM/VL coordination strategies in some tasks, no previous studies have examined sex-specific VM/VL coordination strategies during repeated sprint exercise (RSE).

**Methods:**

In this study, asymptomatic young adults (*N* = 39, 19 females) completed a RSE protocol consisting of 10 × 10 s all-out cycling interspersed by 30 s of passive rest. Electromyographic (EMG) signals from the VM and VL muscles were recorded throughout exercise.

**Results:**

VM:VL ratio did not change with fatigue and was not different between the sexes. From sprint 1 to 10, VM-VL onset delay increased from 9.62 to 16.95 ms and from 19.28 to 45.09 ms in males and females, respectively (*p* < 0.001); however, no sex difference was found (*p* = 0.524). Muscle activation amplitude plateaued at different sprint repetitions in males and females while mechanical work plateaued at similar repetitions.

**Discussion:**

These findings suggest that sex differences in the incidence of PFPS may not be influenced by VM/VL muscle coordination as assessed by EMG.

## 1. Introduction

In order to succeed in high-intensity intermittent sport competitions (e.g., rugby, ice hockey, soccer), athletes are required to maintain high levels of performance while moderating the effects of fatigue. Muscle fatigue is exacerbated toward the end of a competitive event (1) and in parallel, a higher incidence of injuries toward the end of a half and/or match has been reported (2, 3). Consequently, it has been hypothesized that these time-related trends in injury risk are associated with muscle fatigue (4, 5). One type of exercise commonly used to examine fatigue mechanisms related to high-intensity intermittent sports is repeated sprint exercise (RSE), defined as sprints that are ≤10 s in duration with recovery periods of ≤60 s between sprints (6). Changes in muscle coordination during RSE may represent a strategy to minimize muscle fatigue (7). However, it remains unclear how effective these are in reducing injury risk, specifically to knee injuries and conditions that present sex differences. For example, a higher prevalence of patellofemoral pain syndrome (PFPS) has been reported in females compared to males (8, 9). While it has been suggested that anatomical factors such as a greater quadriceps angle may increase the risk for PFPS, literature to support this claim is limited (10). Biomechanical factors such as muscle coordination patterns may be better predictors of PFPS. For example, a lower activation ratio of the vastus medialis (VM) relative to the vastus lateralis (VL) muscle has been observed in patients with PFPS (11, 12). Furthermore, the delayed onset of electromyographic (EMG) activity of the VM relative to the VL is thought to contribute to patellar malalignment and the development of PFPS (13, 14), although sex differences in this parameter have not been studied yet. Therefore, muscle coordination may provide insight into fatigue adaptation strategies as well as key factors contributory to sex-specific injuries and conditions.

To our knowledge, no studies have examined sex differences in VM/VL coordination strategies during RSE, let alone during cycling exercise. However, they have been studied during various other tasks such as knee extension (15), stepping and straight leg raises (16), stair climbing (17), squat (18), and lunge exercises (19). Some of these found that, compared to males, females exhibit a lower VM:VL ratio (19, 20) or delayed onset of VM relative to VL (15). However, some studies have observed no sex differences in VM:VL ratio (16, 18) or vasti onset timing (17). These equivocal results may be explained by several methodological differences between the studies, such as exercise task, normalization procedures, and different calculations of EMG onset timing (e.g., burst onset detection threshold level). Nevertheless, the contrasting findings of these studies warrant further exploration of sex differences in VM/VL coordination patterns, particularly using tasks with greater ecological validity (e.g., RSE).

Therefore, the objective of this study was to compare VM/VL muscle coordination between males and females during RSE. We hypothesized that compared to males, females would generally show: (1) a lower VM:VL ratio and (2) a delayed onset of VM relative to VL with sprint repetitions.

## 2. Methods

### 2.1. Participants

Using the effect size calculated from Torres et al. (19) for sex differences in VM:VL ratio during a lunge exercise, *a priori* sample size calculation (alpha = 0.05, power = 0.80) determined that 38 participants was required. Thus, 20 males (mean ± SD age: 22 ± 2 years, height: 178.9 ± 5.8 cm, mass: 80.7 ± 9.5 kg) and 19 females (age: 22 ± 2 years, height: 166.5 ± 8.4 cm, mass: 64.5 ± 7.6 kg) participated in this study. Participants were included if they had a minimum training duration of 5 h/week, and all participants were healthy with no known neurological or cardiovascular diseases. Female participants were either eumenorrheic (*n* = 13), on monophasic oral contraceptives (>6 months; *n* = 3), or intrauterine device (IUD) users (*n* = 3). Considering the 1st day of the cycle as the onset of menstruation, the eumenorrheic and IUD participants were tested between the 2nd and 5th day of their cycle [i.e., early follicular phase; (21)], while oral contraceptive takers were tested during their 7-day placebo pill phase. This was to control for the estrogen and progesterone levels, thought to affect the neuromuscular function and fatigability of the knee extensors (22). Upon arrival, each participant signed a written informed consent form, completed the Physical Activity Readiness Questionnaire for Everyone (23), and was instructed to refrain from strenuous physical activity, alcohol, caffeine, and food for 48, 24, 12, and 2 h prior to testing, respectively. The study received institutional ethical approval from the McGill University Faculty of Medicine Institutional Review Board (IRB internal study number = A02-B93-20B) and was conducted in accordance with the Declaration of Helsinki.

### 2.2. Experimental design

#### 2.2.1. Electromyography

EMG signals were recorded using wireless surface sensors [Trigno Avanti, Delsys, Natick, MA, USA; double-differential bar (Ag) electrodes; 2.7 cm × 3.7 cm; inter-electrode spacing = 1.0 cm; sampling frequency = 2,000 Hz; common-mode rejection ratio = 80 dB]. Following the SENIAM guidelines (24), sensors were placed on the right VM (4/5 of the distance between the anterior superior iliac spine and the anterior border of the medial collateral ligament of the knee at an orientation nearly perpendicular to the aforementioned line) and VL (2/3 of the distance between the anterior superior iliac spine and the lateral border of the patella at an orientation following this line) muscles. Prior to sensor application, the skin was shaven and abraded with alcohol, and sensors were placed parallel to the muscle fibers.

#### 2.2.2. Maximal voluntary isometric contractions

Maximal voluntary isometric contraction (MVIC) torque of knee extension was measured before and after RSE on an isokinetic dynamometer (CON-TREX MJ, Physiomed Elektromedizin AG, Schnaittach, Germany). The participant sat upright on the dynamometer with the knee fixed at 90° knee flexion, hips and shoulders strapped to the seat, and arms on the side holding on to the seat. Prior to the pre-RSE MVIC, the participant completed a warm-up consisting of isometric knee extensions performed at the following repetitions and relative intensities using the Borg CR10 scale (25): 3 × 3/10; 3 × 5/10; 3 × 7/10, and 2 × 9/10. The participant then performed a MVIC for 3 s while verbal encouragement was provided to ensure maximal effort. EMG and torque data were sampled at 2,000 Hz using a third-party software (Vicon Nexus 2.8.0, VICON Motion Systems Ltd., Oxford, United Kingdom).

#### 2.2.3. Repeated sprint exercise

The participant performed a standardized warm-up involving 5 min of cycling at a comparable wattage of 60–90 W on a mechanically braked cycle ergometer (Monark 894E, Varberg, Sweden). As it is well established that males produce more power than females during cycling (26), females and males performed the warm-up with a load of 1 kg and 1.5 kg, respectively. After 2 min of rest, they performed the RSE protocol which consisted of 10 × 10 s sprints, with the resistance set at 0.075 kg per kg of body mass, interspersed by 30 s of passive rest. Seat height was standardized such that the knee was flexed 15° at the bottom of each stroke. Handlebars were adjusted to the participant's preference, and the cycle ergometer was equipped with toe clips to prevent the participant's feet from slipping. Prior to the start of each sprint (i.e., when the resistance was applied to the flywheel), there was a 3 s acceleration phase where the participant pedaled as fast as they could against no resistance. The participant remained seated for the entire duration of each sprint and rest period in order to optimize reliability and minimize interindividual variability across participants. To ensure that performance and motivation were maximized during each sprint, we adhered to a similar protocol as Hureau et al. (27): (1) the participant was systematically instructed to pedal as fast as possible for the entire duration of the sprint, independent of sprint repetition number, (2) uniform and strong verbal encouragements were provided by the same researcher who was in attendance at every testing session, (3) the participant was naive to the precise purpose of the project and the hypothesized outcomes. More specifically, the participant was told: “Pedal as fast as possible for the entire duration of the sprint, independent of sprint number. There will be a 3 s acceleration phase before each sprint. Loud verbal encouragements will be given. During rest periods between sprints, you are not allowed to move. Do not leave the seat.” Computer software (Monark Anaerobic Test Software) was used to record the raw cycle ergometer data and analyzed offline.

### 2.3. Data analysis

All EMG data were band-pass filtered (Butterworth 3rd order, 10–500 Hz), full-wave rectified, and root mean squared (RMS) with a 25-ms moving window (7). A threshold level of 35% of the mean EMG RMS value of each sprint was used to determine EMG burst onset, based on previous literature (28). Each EMG burst was visually inspected to confirm timing identification for each burst. To quantify the relative difference in EMG onset timing of the VM and VL (VM−VL onset delay), the onset of the VL signal was subtracted from that of the VM signal for each burst within each sprint. The mean onset delay was then calculated for each sprint. A positive value implies that the VL burst onset occurred first, and a negative value implies that the VM burst onset occurred first. To calculate the RMS values for each sprint, bursts were concatenated (i.e., time periods between bursts where the EMG signal did not attain the threshold value were removed) to produce one continuous series of bursts per sprint (27). Within each sprint, the mean RMS value was calculated for each muscle (RMS_VM_; RMS_VL_) and normalized to the first sprint's RMS as previous RSE studies have used a dynamic normalization method where EMG values are expressed relative to peak or mean initial sprint value (29, 30, 31). To calculate the VM:VL ratio for each sprint, RMS_VM_ was divided by RMS_VL_ before normalizing it to the first sprint's VM:VL ratio.

The raw data from the Monark Anaerobic Test Software included timestamp data acquired every time a magnet on the flywheel passed the sensor. Cadence (rpm) was extracted from the raw data. Using a previously described method (32), instantaneous power output during sprints was quantified from the flywheel velocity and corrected for the effect of inertia. Mechanical work output (J) for each sprint was calculated by integrating the power curve over the 10 s sprint using the trapezoidal rule. Using the same procedure as Fitzsimons et al. (33), fatigability (i.e., % decrement; Equation 1) was calculated.

Equation 1. Calculation of % decrement (33). Work_ideal_ refers to ideal work (i.e., highest mechanical work across all sprints multiplied by the total number of sprints in RSE protocol); Work_total_ refers to total work (i.e., sum of mechanical work performed during all 10 sprints).

$$\text{\%}\;\;\mathrm{decrement}=100-\left(\frac{\mathrm{Wor}k_{\mathrm{total}}}{\mathrm{Wor}k_{\mathrm{ideal}}}\times 100\right)$$

### 2.4. Statistical analysis

Data are presented as means ± SD in the tables and as means ± SE in the figures. Independent *t*-tests were conducted to compare Worktotal and % decrement between the sexes. Normal Gaussian distribution of data was confirmed via Shapiro–Wilk test. If there was a violation, data were transformed within the generalized estimated equations (GEEs) using a logarithmic Link Function. MVIC torque, was tested for main and interaction effects of Condition (pre-exercise, post-exercise) and Sex (male, female) via GEEs. VM:VL ratio, VM−VL onset delay, mechanical work, and cadence were tested for main and interaction effects of Sprint (1–10) and Sex (male, female) via GEEs. RMS was tested for main and interaction effects of Sprint (1–10), Sex (male, female), and Muscle (VM, VL) via GEEs. For MVIC torque, VM:VL ratio, mechanical work, cadence, and RMS, “Identity” Link Function was used, while for VM−VL onset delay, “Log” Link Function was used. A first-order autoregressive (AR1)” correlation structure was used based on best goodness of fit, evaluated as the lowest quasi-likelihood under the independence model criterion (i.e., QIC) statistic. For statistically significant effects, specific pairwise comparisons (Wald X2) with sequential Bonferroni correction were performed. The statistical significance was set at *p* < 0.05 and all statistical analyses were performed in SPSS (v23, IBM Corporation).

## 3. Results

### 3.1. Mechanical work, torque, and cadence

Group cycling and strength characteristics are given in Table 1. Work_total_ (*p* < 0.001) and % decrement (*p* = 0.016) were greater in males compared to females. MVIC torque decreased more from pre-exercise to post-exercise in males compared to females (Sex × Condition interaction: *X*^2^ = 43.050, *p* < 0.001).

**Table 1:** Participant and performance characteristics.

	Males	Females	*P* value
*N*	20	19	n/a
Age (years)	22 ± 2	22 ± 2	0.500
Height (cm)	178.9 ± 5.8	166.5 ± 8.4	**<0** **.** **001**
Body mass (kg)	80.7 ± 9.5	64.5 ± 7.6	**<0** **.** **001**
Pre-exercise MVIC torque (Nm)	262.5 ± 52.6	164.0 ± 31.6	**<0** **.** **001**
Post-exercise MVIC torque (Nm)	161.3 ± 44.8	115.0 ± 30.5	**0** **.** **001**
Mechanical work			
Work_total_ (kJ)	50.9 ± 8.3	38.2 ± 8.5	**<0** **.** **001**
% decrement (%)	33.1 ± 8.0	26.0 ± 9.5	**0** **.** **016**

Bold values indicate *p* values smaller than 0.05.

**Figure 1:**
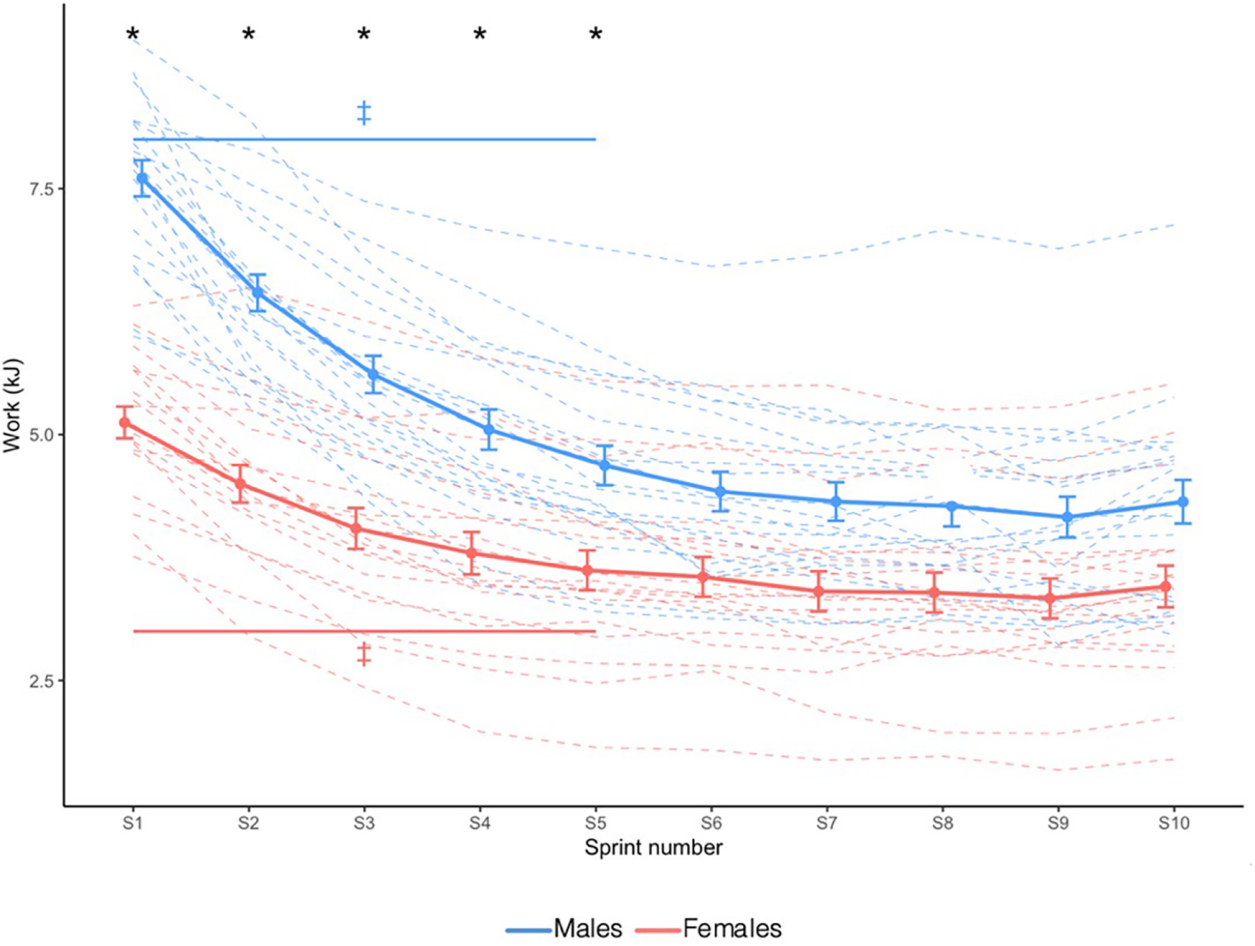
Absolute mechanical work. Dashed lines indicate individual participants and solid lines indicate group mean. Error bars represent standard error. * Greater in males than females (*p *< 0.05). ‡ Significantly different from sprint 10 (Sex × Time interaction effect; *p *< 0.05).

Absolute mechanical work is shown in Figure 1. In both sexes, mechanical work decreased until sprint 6 and plateaued; however, males produced greater mechanical work than females only during the first five sprints (Sex × Sprint interaction: *X*^2^ = 23.409, *p* = 0.005).

**Figure 2:**
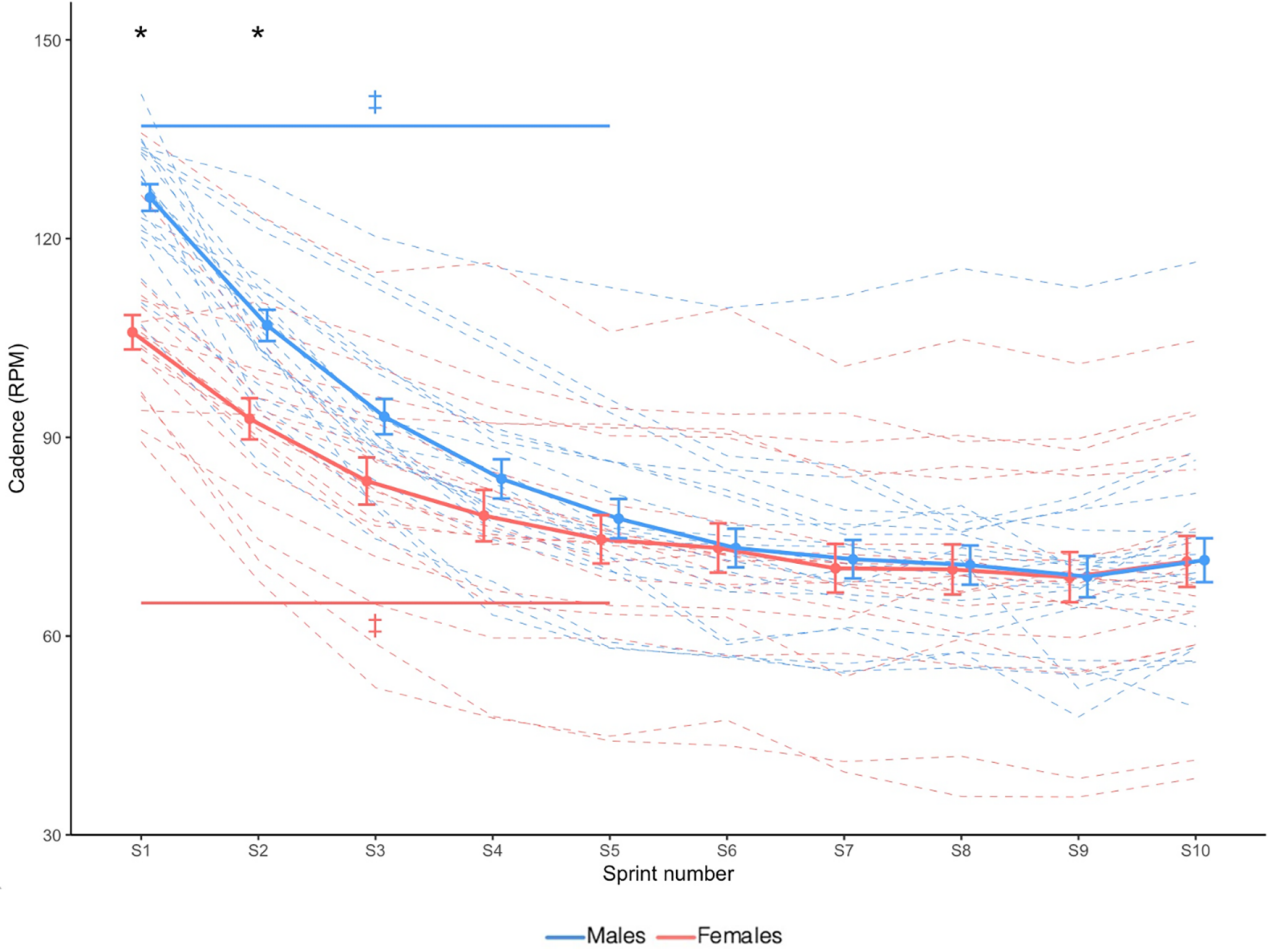
Cadence. Dashed lines indicate individual participants and solid lines indicate group mean. Error bars represent standard error. * Greater in males than females (*p *< 0.05). ‡ Significantly different from sprint 10 (Sex × Time interaction effect; *p *< 0.05).

Cadence is shown in Figure 2. Sex × Sprint interaction effect revealed that compared to females, males had a greater cadence only during sprint 1 and sprint 2 (*X*^2^ = 44.917, *p* < 0.001).

### 3.2. Muscle activation amplitude

**Figure 3:**
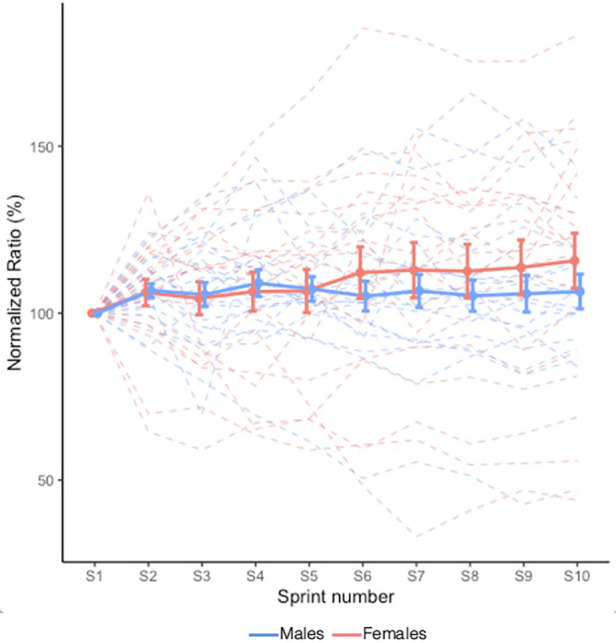
Mean VM:VL ratio during RSE. Values are normalized to the initial sprint value. Dashed lines indicate individual participants and solid lines indicate group mean. Error bars represent standard error. A value greater than 100.0% represents a greater activation of VM relative to VL.

VM:VL ratio is shown in Figure 3. There were no Sex × Sprint interaction (*X*^2^ = 9.322, *p* = 0.408) nor main Sprint (*X*^2^ = 14.361, *p* = 0.110) or Sex (*X*^2^ = 0.368, *p* = 0.544) effects.

**Figure 4:**
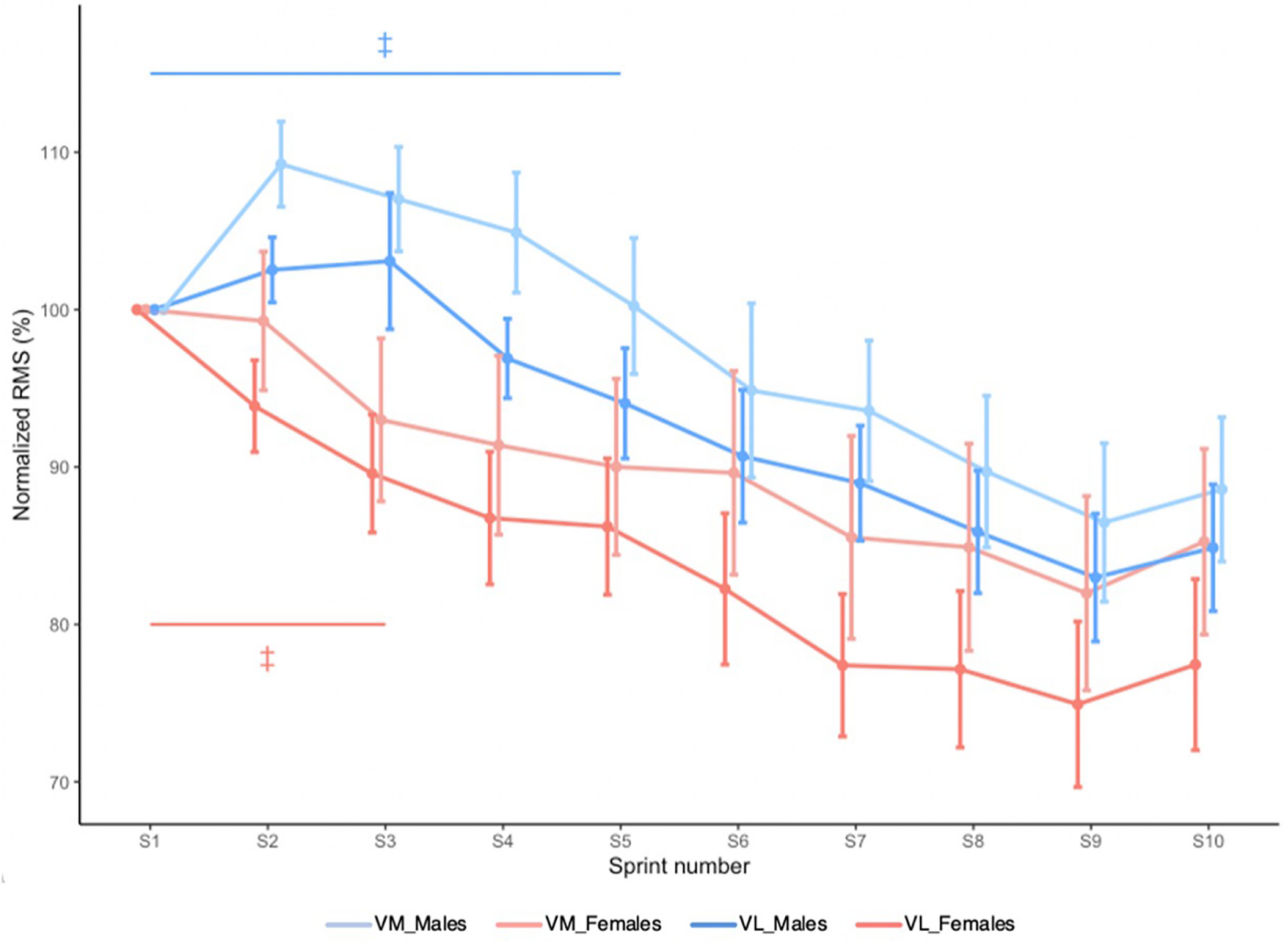
Mean RMS_VM_ and mean RMS_VL_ during RSE. Values are normalized to the initial sprint value. Error bars represent standard error. ‡ Significantly different from sprint 10 (Sex × Time interaction effect; *p *< 0.05).

RMS_VM_ and RMS_VL_ are shown in Figure 4. There was no Sex × Sprint × Muscle interaction effect (*X*^2^ = 11.490, *p* = 0.244). Sex × Sprint interaction effect revealed that in males, knee extensor RMS decreased until sprint 6 and then plateaued, while in females, it decreased until sprint 4 and then plateaued (*X*^2^ = 20.043, p = 0.018).

### 3.3. Muscle activation timing

**Figure 5:**
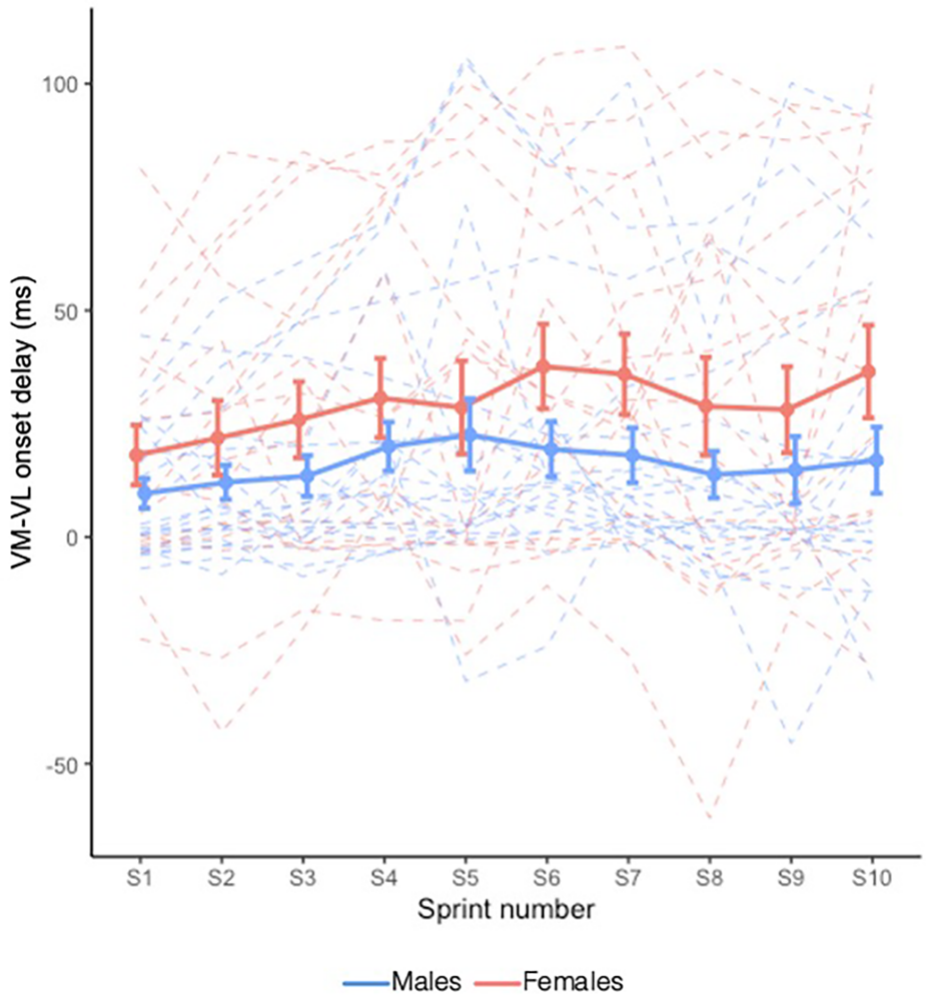
Mean VM−VL onset delay during RSE. Dashed lines indicate individual participants and solid lines indicate group mean. Error bars represent standard error.

VM−VL onset delay is shown in Figure 5. VM−VL onset delay generally increased with sprints (*X*^2^ = 31.993, *p* < 0.001); however, this occurred independently of Sex (i.e., no Sex × Sprint interaction effect: *X*^2^ = 8.106, *p* = 0.524). No main effect of Sex was observed (*X*^2^ = 2.023, *p* = 0.155).

## 4. Discussion

The main findings of the present study were: (1) VM:VL ratio remained stable during RSE while the time delay between VM and VL activation increased, (2) there were no sex differences in VM:VL ratio and VM−VL onset delay during RSE, suggesting similar VM/VL coordination strategies between the sexes, and (3) muscle activation amplitude and mechanical work plateaued at similar sprint repetition in males, while in females, muscle activation amplitude plateaued before mechanical work output.

### 4.1. Effects of fatigue

MVIC torque and mechanical work were significantly reduced with exercise, confirming the presence of muscle fatigue. Both RMS_VM_ and RMS_VL_ decreased with fatigue, in line with previous studies examining EMG activation amplitude during all-out cycling (29, 31, 34). This decrease may result from reduced neural drive to the active musculature and represent a protective mechanism mediated by group III and IV muscle afferents (35, 36). Furthermore, RMS_VM_ and RMS_VL_ decreased similarly with fatigue, and accordingly, no change in VM:VL ratio was observed. Similarly, Myer et al. (20) observed no main effect of repetition number on VM:VL ratio during a dynamic knee exercise, suggesting that VM:VL ratio is not affected by fatigue.

On the contrary, we found that VM−VL onset delay generally increased with fatigue, indicating that with fatigue, there was a longer delay in the activation of VM relative to VL. A delayed onset of VM relative to VL has been theorized to result in an imbalance of medial-to-lateral force acting at the knee joint (37) and contribute to the development of PFPS (14). However, this theory assumes a direct relationship between EMG signal and muscle force, and does not account for the electromechanical delay. Alternatively, changes in muscle coordination during RSE have been thought to contribute to reductions in power output and act as a preventative measure to limit peripheral muscle damage (7). Thus, while the observed increase in VM−VL onset delay may reflect a preventative mechanism to muscle fatigue, it could also come with a cost of increasing the risk for PFPS.

### 4.2. No sex differences in muscle coordination

Contrary to our hypotheses, we found that VM:VL ratio and VM−VL onset delay did not differ between the sexes. To our knowledge, this is the first study to compare VM:VL ratio and VM−VL onset delay between females and males during RSE. Using different exercise modalities, previous studies have reported mixed sex comparison results. Unlike our findings, a significantly lower VM:VL ratio has been reported in females during a task involving the participant to flex and lean on the examined knee (20) and during a lunge and Bulgarian squat exercise (19). Our results are, however, in agreement with those of Bowyer et al. (16) who found no sex differences in VM:VL ratio during a stepping activity and straight leg raise, and those of Nimphius et al. (18) who found no sex differences in VM:VL ratio during an isometric squat. Furthermore, Cowan and Crossley (17) reported no sex differences in VM−VL onset delay during a dynamic-stepping task. PFPS is more prevalent in females than males (8, 9) and it has been thought that an imbalance of force amplitude and timing between the VM and VL represents a critical risk factor. However, muscle force cannot be inferred from neural drive alone as it also depends on other biomechanical factors such as specific tension (defined as maximal force per unit area) and physiological cross-sectional area [defined as the area of a muscle perpendicular to its fibers; (38)]. Similarly, a delayed onset of EMG activation does not directly imply delayed force production but may instead represent a delay in myoelectric activity under the recording electrode, or electromechanical delay, which reflects both electrochemical processes such as synaptic transmission and excitation-contraction coupling, and mechanical processes like force transmission (38). As such, it is speculated that sex differences in the incidence of PFPS may not be directly related to VM/VL muscle coordination as assessed by EMG. Therefore, rather than imbalance of EMG activation amplitude and delayed EMG activation, future studies are warranted to examine the force imbalances and timing differences in force production. While physiological cross-sectional area can be measured using magnetic resonance imaging (39), specific tension is more difficult to estimate and therefore measurement of muscle force remains a challenge (38). However, timing differences in force production may be estimated by using ultrafast ultrasound sampling (40). This is to determine whether there is an association between delayed EMG activation and delayed muscle/patellar motion, as well as to quantify the delay between VM and VL motion onset (38).

### 4.3. Differing plateau timing of muscle activation amplitude and mechanical work

During RSE, strong correlations between decline in mechanical work and EMG activation amplitude have been reported (29, 31, 34, 41), suggesting that EMG activation amplitude may be an index of central motor drive. In our study, while knee extensor muscle activation amplitude and mechanical work plateaued at similar sprint repetitions (sprint 6) in males, females showed earlier plateauing of muscle activation amplitude (sprint 4) but a continued decrease in mechanical work (up to sprint 6). It is well established that females generally have a higher proportion of type I fibers than males (42) and type I fibers may be deactivated earlier than type II fibers at high contraction frequencies (43), such as those required during RSE. This may explain why muscle activation amplitude plateaued at different times between the sexes. Further, the discrepancy in plateau timing between muscle activation and mechanical work in females may be elucidated by examining other muscles than just VM and VL. Previously, Billaut and Smith (29) reported that knee extensor activity differed between sexes during RSE while biceps femoris (i.e., knee flexor) muscle activity did not. Therefore, we speculate that other muscles, such as the biceps femoris, may compensate for knee extensor muscle fatigue in the latter portion of RSE in females specifically. Rapid movements produced by coordinated extensor and flexor activity may be particularly disrupted by fatigue (44). Although unexamined in the present study, the dyscoordination between knee extensors and flexors in the latter portion of RSE may be related to the greater injury risk in females (e.g., PFPS), or rather, more injury-protective mechanisms in this injury-prone population.

## 5. Limitations

This study exhibited a number of limitations. Firstly, it has recently been reported that errors in VM−VL onset may amount to −12 ms when not taking the relative position of bipolar electrodes between muscles into account (45). Therefore, it is possible that sex differences in onset timing did not emerge due to possible discrepancies in the position of bipolar electrodes in relation to the innervation zone for VM and VL. In addition, local EMG detection assumes accurate representation of the target muscle and no other muscles. We acknowledge that this assumption may result in Type II error (46), especially given the documented evidence of regional differences within and between the VM and VL (47). Next, if there are indeed no sex differences in VM/VL coordination strategies as results indicate, other factors such as force imbalances or quadriceps angle may be more relevant to help explain the higher prevalence of PFPS in females compared with males. As well, concurrent analysis of knee flexor EMG activity could provide a more comprehensive overview to muscle coordination strategies during RSE. Additionally, it has been reported that exercise performance is greater during the luteal phase compared to the follicular phase (22), although we speculate that the effect of menstrual cycle phase on muscle fatigue is moderate compared to the effect of biological sex. Lastly, in a portion of our participants, we concomitantly measured muscle oxygenation using a near infra-red spectroscopy probe placed on the same leg; however, since a similar number of male and female participants were tested under this condition, and since the probe was in place for the duration of the fatiguing protocol, we estimate that its effect would have been constant across sexes and throughout RSE protocol times, such that it likely did not affect the statistical results presented here.

## 6. Conclusions

This study compared VM/VL coordination strategies during RSE between males and females. There were no sex differences in VM:VL ratio and VM−VL onset delay; however, muscle activation amplitude plateaued before mechanical work in females. It is postulated that other muscles (e.g., knee flexors) may compensate for the plateauing of knee extensor activation to maintain mechanical work output in females. Thus, while VM/VL coordination was comparable between the sexes, coordination with other muscle groups may differ between males and females, which could help elucidate the mechanisms contributory to females’ greater risk of PFPS.

## Data Availability

The raw data supporting the conclusions of this article will be made available by the authors, without undue reservation.
